# Immune System-Related Changes in Preclinical GL261 Glioblastoma under TMZ Treatment: Explaining MRSI-Based Nosological Imaging Findings with RT-PCR Analyses

**DOI:** 10.3390/cancers13112663

**Published:** 2021-05-28

**Authors:** Pilar Calero-Pérez, Shuang Wu, Carles Arús, Ana Paula Candiota

**Affiliations:** 1 Departament de Bioquímica i Biologia Molecular, Unitat de Bioquímica de Biociències, Edifici Cs, Universitat Autònoma de Barcelona, 08193 Cerdanyola del Vallès, Spain; Pilar.Calero@uab.cat (P.C.-P.); psychews@gmail.com (S.W.); Carles.Arus@uab.cat (C.A.); 2 Centro de Investigación Biomédica en Red en Bioingeniería, Biomateriales y Nanomedicina (CIBER-BBN), 08193 Cerdanyola del Vallès, Spain; 3 Institut de Biotecnologia i de Biomedicina (IBB), Universitat Autònoma de Barcelona, 08193 Cerdanyola del Vallès, Spain

**Keywords:** glioblastoma, orthotopic immunocompetent tumours, immune-enhancing metronomic schedule, TMZ, magnetic resonance spectroscopic imaging, immune system activity imaging biomarker, cancer immune cycle, glioma-associated microglia/macrophages, PD-L1

## Abstract

**Simple Summary:**

Glioblastoma (GB) is an aggressive brain tumour with poor survival. Tumour microenvironment is a key element in GB evolution and response to therapy. We assessed presence and phenotypes of microglia/macrophages in preclinical GL261-GB microenvironment under Temozolomide (TMZ) treatment to unveil its possible relationship with MRSI-detected metabolomics changes. Microglia/macrophage polarisation towards an anti-tumour phenotype prevailed in TMZ-treated tumours. Since microglia/macrophages can represent 30–40% of the GB tumour volume, they must contribute the metabolomic pattern change. PD-L1 expression also correlated with the anti-tumour microglia/macrophage phenotype. These results highlight the potential of MRSI-detected metabolomics as non-invasive biomarker for immune system action.

**Abstract:**

Glioblastomas (GB) are brain tumours with poor prognosis even after aggressive therapy. Previous work suggests that magnetic resonance spectroscopic imaging (MRSI) could act as a biomarker of efficient immune system attack onto GB, presenting oscillatory changes. Glioma-associated microglia/macrophages (GAMs) constitute the most abundant non-tumour cell type within the GB and can be polarised into anti-tumour (M1) or pro-tumour (M2) phenotypes. One of the mechanisms to mediate immunosuppression in brain tumours is the interaction between programmed cell death-1 ligand 1 (PD-L1) and programmed cell death-1 receptor (PD-1). We evaluated the subpopulations of GAMs in responding and control GB tumours to correlate PD-L1 expression to GAM polarisation in order to explain/validate MRSI-detected findings. Mice were evaluated by MRI/MRSI to assess the extent of response to treatment and with qPCR for GAMs M1 and M2 polarisation analyses. M1/M2 ratios and PD-L1 expression were higher in treated compared to control tumours. Furthermore, PD-L1 expression was positively correlated with the M1/M2 ratio. The oscillatory change in the GAMs prevailing population could be one of the key causes for the differential MRSI-detected pattern, allowing this to act as immune system activity biomarker in future work.

## 1. Introduction

Glioblastoma (GB) is the most frequent primary central nervous system malignancy in adults. These tumours have poor prognosis, which has not significantly improved despite new diagnostic strategies and innovative therapies have been developed [1]. The combination of chemotherapy (Temozolomide, TMZ) plus radiotherapy is still used as the standard therapeutic choice after surgery, resulting in an average survival rate of only 14.6 mo [2], which highlights the urgent need for investigating novel therapeutic approaches/follow-up strategies in order to improve patient outcome. Nowadays, the implication of the immune system in cancer surveillance and therapy response is widely accepted [3]. This is especially relevant in GB, since GB cells have the capacity for creating an immunosuppressive microenvironment and employ various methods to escape immune surveillance through several pathways [4]. Therefore, understanding these strategies and the biology of such tumour microenvironment will be helpful for developing novel therapeutic approaches and follow-up methods, which should lead to improved prognosis for GB patients.

Glioma-associated microglia/macrophages (GAMs), i.e., microglia, together with peripheral macrophages recruited by tumour tissue from circulating blood [5], constitute the most common non-tumour cell type in the GB microenvironment [6] and are characterised by considerable diversity and plasticity. Over the last decade, it has become clear that this cellular population interacts with numerous other cell types to actively influence brain tumour biology [7]. These cells can be activated by various stimuli and polarised into classically activated (M1) or alternatively activated (M2) phenotype, which represent extreme situations of a continuum of activation states. M1 microglia/macrophages are usually involved in proinflammatory and anti-tumour mechanisms. In contrast, M2 microglia/macrophages are involved in activities for promoting tumour survival and growth [8]. Accordingly, their gradual polarisation state determines the pathophysiological character of this cell population, while subpopulations differ with respect to receptor expression, effector function, and cytokine and chemokine production. The antitumor effect of M1 microglia/macrophages was described to involve several mechanisms, such proinflammatory cytokines release and activation of cytotoxic T lymphocytes. Indeed, complex interactions between innate and adaptive immune responses such as antitumour microglia/macrophages and T-cells have been described [9] and are key elements in sustained therapy response. On the other hand, M2 microglia/macrophages can increase the proportion of T-regulatory lymphocytes and lead to the inhibition of the cytotoxic T-cell responses [10]. The fact that microglia/macrophages had been described to be more abundant in GB than in low-grade gliomas [6] hints at their possible active role in glioma progression. GAMs undergoing phenotype polarisation display changes in their molecular and metabolic profiles, also triggering the expression of different markers such as Nos2 and CD206 characterising M1 and M2 microglia/macrophages, respectively [11,12]. However, more than a bilateral all or none option, the M1/M2 signature rather implies a continuum between two extremes with specific abilities (e.g., epigenetic marks, metabolic reprogramming) and local signalling (e.g., cytokines, chemokines, and immune checkpoints) [13].

Furthermore, one of the main mechanisms known to mediate immunosuppression in the brain tumour microenvironment is the interaction between programmed cell death-1 ligand 1 (PD-L1) and its receptor (PD-1) [14]. PD-1 is an inhibitory receptor mainly expressed on activated T cells, B cells, macrophages, and dendritic cells, while PD-L1 is highly expressed by malignant tumour cells [15], as well as by tumour-infiltrating myeloid cells, including macrophages [16,17]. It is a notable immune checkpoint initially described to cause T cell anergy [18], although its role in other cellular populations is still a matter of discussion.

The preclinical GB model GL261 growing into C57BL/6 mice is widely accepted as an immunocompetent model suitable for assessing therapeutic approaches and immune system participation in therapy response [9,19,20,21]. Our group has studied the evolution and behaviour of this tumour model under TMZ and other therapeutic approaches [22,23,24,25]. We have described the non-invasive assessment of response to therapy in preclinical GL261 GB (control and under TMZ therapy) with magnetic resonance spectroscopic imaging (MRSI) approaches. MRSI combines anatomical information from magnetic resonance imaging (MRI) [26], and magnetic resonance spectroscopy (MRS), which provides information regarding the metabolomic profile of the investigated tissue [27,28,29]. When using MRSI is coupled to advanced machine learning analysis (source extraction as described in [30]), spectral pattern differences between actively proliferating GB and GB responding to therapy can be shown colour-coded (the nosological images) in single-slice [23] or multislice volumetric approaches [24]. Using volumetric MRSI-based nosological images, we have defined the tumour responding index (TRI) as an evaluation parameter to estimate/measure the extent of response to treatment. The level of detected response, TRI, showed consistent oscillations with 6–7 day frequency during transient response to TMZ therapy [24,31]. These oscillations were not detected regarding tumour volume changes. We hypothesised that this could be a surrogate biomarker for immune system-contributed local changes triggered by response to therapy, since this periodicity coincides with the cancer immune cycle length described in [32], also supported by immunohistochemistry data related to lymphocytes (CD3^+^) and microglia/macrophage (Iba1^+^) content [31]. 

The purpose of this work was to assess the origin of the oscillating pattern changes spotted by MRSI in TMZ-treated/responding GL261 GB bearing mice under Immune-Enhancing Metronomic Schedule (IMS) [31]. Since it was described that macrophages can represent up to 30–40% of tumour masses [5], consistent with our results in [31], it is reasonable to investigate whether the spectral pattern changes could be related to different macrophage phenotypes present in such samples. Accordingly, we wanted to characterise the different subpopulations of GAMs in MRSI-evaluated responding and control GL261 GB tumours by quantitative real-time polymerase chain reaction (qPCR). Furthermore, PD-L1 gene expression was investigated by qPCR to correlate GAMs polarisation with immunosuppression within the tumour microenvironment. 

## 2. Materials and Methods

### 2.1. GL261 GB Preclinical Model Generation and Treatment

GL261 mouse glioma cells have been obtained from the Tumour Bank Repository at the National Cancer Institute (Frederick, Maryland) and were grown as previously described by us [33]. Cells were checked for mouse short tandem repeat (STR) profile as well as interspecies contamination. In addition, PCR studies were performed in order to discard mycoplasma and virus presence. All studies involving animals were approved by the local ethics committee (*Comissió d’Ètica en Experimentació Animal i Humana*, CEEAH, www.uab.cat/web/experimentacio-amb-animals/presentacio-1345713724929.html, accessed on 26 May 2021), according to regional and state legislations (protocol references CEA-OH-9685/CEEAH-3665). Mice were purchased from Charles River Laboratories (l’Abresle, France) and housed at the animal facility of the *Universitat Autònoma de Barcelona*. An enriched environment was used, similar to the one described in [34], and mouse spent at least three weeks in this environment prior to tumour implantation. In order to obtain reproducible and well-categorised groups regarding tumour volume and evolution prior to therapy and ensuring proper TRI and tumour volume for qPCR studies (see Section 2.2.4 and Section 2.4), tumours were induced in a total of 110 C57BL/6 female wild-type (wt) mice by intracranial stereotactic injection of 10^5^ GL261 cells as already described by us [33]. Only 20 C57BL/6 mice (weighing 21.1 ± 1.5 g, aged 15.9 ± 3.3 weeks) fulfilling inclusion conditions (e.g., TRI value, equal or higher than 60% for TMZ-treated mice and TRI = 0%, or as close as possible, for control mice, see SM for further details) were eventually used for the evaluation of the origin of the recorded MRSI patterns. Mice were weighed twice a week and tumour volumes were followed up using T2 weighted image (T2w) MRI acquisitions. Multi-slice MRSI studies (Section 2.2) were performed to assess the extent of the response to treatment using the obtained nosological images. Tumour volumes were chosen aiming to ensure enough volume to allow for proper MRSI acquisition and segmentation at the desired time points, with no significant differences between groups.

GL261 GB treatment. For in vivo experiments, TMZ (Sigma-Aldrich, Madrid, Spain) was dissolved in 10% dimethyl sulfoxide (DMSO) in saline solution (0.9% NaCl) and was administered using an oral gavage. Treated tumour-bearing mice (*n* = 10) received IMS-TMZ 60 mg/kg, every 6 d (two or three times depending on the euthanasia day), from day 11 post-implantation (p.i.), as described in [31], while tumour-bearing control mice (*n* = 10) received 10% DMSO vehicle following the same administration schedule.

### 2.2. In Vivo MRI and MRSI Studies

In vivo MRI/MRSI studies were conducted at the joint nuclear magnetic resonance facility of the *Universitat Autònoma de Barcelona* and *Centro de Investigación Biomédica en Red-Bioingeniería, Biomateriales y Nanomedicina* (CIBER-BBN) (Cerdanyola del Vallès, Spain), Unit 25 of NANBIOSIS (www.nanbiosis.es, accessed on 18 March 2021). Mice were positioned in a dedicated bed, which allowed suitable anaesthesia delivery (isoflurane, 1.5–2.0% in O_2_ at 1 L/min), with an integrated circuit of heating water for maintaining proper body temperature. Respiratory frequency was monitored with the help of a pressure probe and kept between 60–80 breaths/min. The 7T Bruker BioSpec 70/30 USR spectrometer (Bruker BioSpin GmbH, Ettlingen, Germany) equipped with a mini-imaging gradient set (400 mT/m) was used for acquisitions. A 72-mm inner-diameter linear volume coil was used as transmitter, and a dedicated mouse brain quadrature surface coil was used as receiver for MRI studies. 

#### 2.2.1. MRI Studies

GL261 GB-bearing mice were screened with high-resolution coronal T2w images using a Rapid Acquisition with Relaxation Enhancement (RARE) sequence to evaluate brain tumour presence and to monitor its evolution stage, using repetition time (TR)/effective echo time (TEeff) = 4200/36 ms. The detailed set of parameters used in MRI acquisitions can be found in Supplementary Material file. MRI data of tumour-bearing mice were acquired and processed on a Red Hat Linux computer using ParaVision 5.1 software (Bruker BioSpin GmbH, Ettlingen, Germany).

#### 2.2.2. MRSI Studies

Consecutive point-resolved spectroscopy (PRESS), 14 ms echo time (TE) MRSI were acquired individually across the tumour, using T2w high-resolution images as reference, as described in [24]. Shimming was individually performed for each MRSI grid. MRSI grids were spatially placed in order that the volume of interest (VOI) would include most of the tumoral mass as well as part of the normal/peritumoral brain parenchyma. The whole set of MRSI acquisition parameters can be found in the Supplementary Material file.

#### 2.2.3. MRI and MRSI Processing and Post-Processing

- Tumour volume calculation

Abnormal brain masses observed in T2w images were manually segmented and each tumour volume was calculated from T2w high-resolution horizontal images using the following equation:

TV mm^3^ = [(AS1 × ST) + [(AS2 + (…) + AS10) × (ST + IT)]] × 0.0752 (1)

where TV is the tumour volume, AS is the number of pixels in the region of interest in each MRI slice, ST is slice thickness, IT the inter-slice thickness, and 0.0752 accounts for the individual pixel surface area in mm^2^. The tumour area was calculated from pixels in each slice with ParaVision 5.1 software. The inter-slice volume was estimated through addition of the inter-slice thickness to the corresponding slice thickness in Equation (1).

- Brain MRSI post-processing and machine learning strategies

MRSI data were post-processed as described by us in [35]. Briefly, data were initially pre-processed with ParaVision 5.1. Further post-processing was performed with 3D Interactive Chemical Shift Imaging (3DiCSI) software package version 1.9.17 (Courtesy of Truman Brown, PhD, Columbia University, New York, NY, USA) for the following: line broadening adjustment (Lorentzian filter, 4 Hz), zero-order phase correction, and ASCII format exportation. Then, dynamic MRSI processing module (DMPM, http://gabrmn.uab.es/?q=dmpm, accessed on 26 May 2021), running over MatLab 2013a (The MathWorks Inc., Natick, MA, USA), was used for spectral alignment within each MRSI matrix, using the 3.21 ppm choline signal as reference). The 0–4.5 ppm region of each MRSI spectrum was unit length normalised and exported in ASCII format used for pattern recognition (PR) analysis. No baseline correction was performed in these spectra. 

After that, spectral vectors were analysed following the methodology based in non-negative matrix factorization (NMF) semi-supervised protocol described in [23] for classifying pixels into normal brain parenchyma, actively proliferating tumour and tumour responding to treatment, and calculating nosologic maps representing the spatial response to treatment. Green colour is used when the GB responding to treatment source contributes the most, red for actively proliferating GB, blue for normal brain parenchyma, and black for undetermined tissue. See Section 4.3 for further details about metabolites originating the differences in the control or responding GB pattern metabolome (also SM and Figure S1).

#### 2.2.4. Tumour Responding index (TRI) Calculations

In order to measure the extent of response to treatment using the obtained nosological images, a numerical parameter named TRI was calculated (Equation (2)) [24],

(2) $$\mathrm{TRI}=\frac{Tumour\;responding\;pixels}{Total\;tumour\;pixels}\times 100$$

TRI is stated as the percentage of green (colour-coded), responding tumour pixels of all grids over the total tumour pixels of all recorded grids. 

Then, extreme values for TRI were selected, as homogeneously green/red as possible. For TMZ-treated mice, tumours with TRI values >60% were selected while TRI = 0% (or as close as possible) was selected for vehicle-treated mice. Regarding cases responding to TMZ treatment, tumour volume meeting criteria for “stable disease” according to Response Evaluation Criteria in Solid Tumors (RECIST) [36] adapted as described in [23] were chosen. 

### 2.3. Animal Euthanasia

Whenever a mouse met criteria to be included in the study regarding MRI and MRSI parameters, euthanasia was performed by cervical dislocation, and samples were dissected and stored in liquid nitrogen.

### 2.4. RNA Isolation, cDNA Synthesis and qPCR

RNA isolation was performed following the protocol for purification of total RNA from animal tissues (RNeasy Mini Kit, QIAGEN, GmbH, Hilden, Germany). RNA concentration was quantified at 260 nm (Qubit, Thermo Fisher Scientific, Massachusetts, EEUU), and RNA integrity and quality were determined with 260/280 and 260/230 ratios (NanoDrop, München, Germany). One hundred ng of RNA from each sample was transcribed into cDNA with the iScript cDNA synthesis kit (Bio-Rad, California, EEUU) according to manufacturer’s instructions.

The qPCR amplification was carried out to investigate five genes: EGF-like module containing mucin-like hormone receptor-like 1 (F4/80), inducible NO synthase (Nos2), mannose receptor C type 1 (CD206) programmed cell death-1 ligand 1 PD-L1 (CD274 antigen), purchased from BioRad (California, EEUU, Ref 13,733; 18,126; 17,533; and 60,533, respectively). F4/80 has been established as a global microglia/macrophage population marker, Nos2 has been described as a marker of M1 phenotype, and CD206 has been defined as a marker of M2 phenotype [37,38,39,40].

An amount of 2 ng of cDNA was used for qPCR, all reactions were performed twice, and results were averaged. qPCR analysis was carried out using the Bio-Rad CFX qPCR System. Primers and the Power SYBR Green Master Mix were purchased from Bio-Rad. The PCR amplification reactions were performed in 10 μL reaction volumes, and PCR protocol consisted of 40 cycles of denaturation at 95 °C for 10 s and annealing/extension at 60 °C for 30 s. Relative mRNA expression levels were normalised to two housekeeping genes (tata binding protein (TBP) and hypoxanthine guanine phosphoribosyl transferase (HPRT), purchased from BioRad (California, EEUU, Ref 21,374 and 15,452, respectively). Primers sequences are described in the Supplementary Material file. The cycles threshold-values (ct-values) average of the two reference genes for normalization purposes was used. For each gene, the 2^−ΔΔCt^ method [41] (a method to calculate relative gene expression levels between different samples that directly uses the ct-values generated by the qPCR system) for calculation was performed to analyse relative quantities.

### 2.5. Statistical Analysis

Sample distribution was assessed with Kolmogorov-Smirnov test. Levene’s test was used for assessing variance homogeneity. A two-tailed Student’s t-test for independent measurements was used for comparisons. Relationships between different markers were assessed with the Pearson correlation coefficient. The significance level for all tests was *p* < 0.05. 

## 3. Results

### 3.1. Follow up of GL261 Tumour-Bearing Mice and Endpoint Criteria

In this study, 20 mice were used (*n* = 10 IMS-TMZ-treated and *n* = 10 vehicle-treated). The average tumour volume at therapy start (day 11 p.i.) was 7.5 ± 3.2 mm^3^ for TMZ-treated mice and 9.1 ± 8.4 mm^3^ for control mice, with no significant differences between both groups.

**Figure 1:**
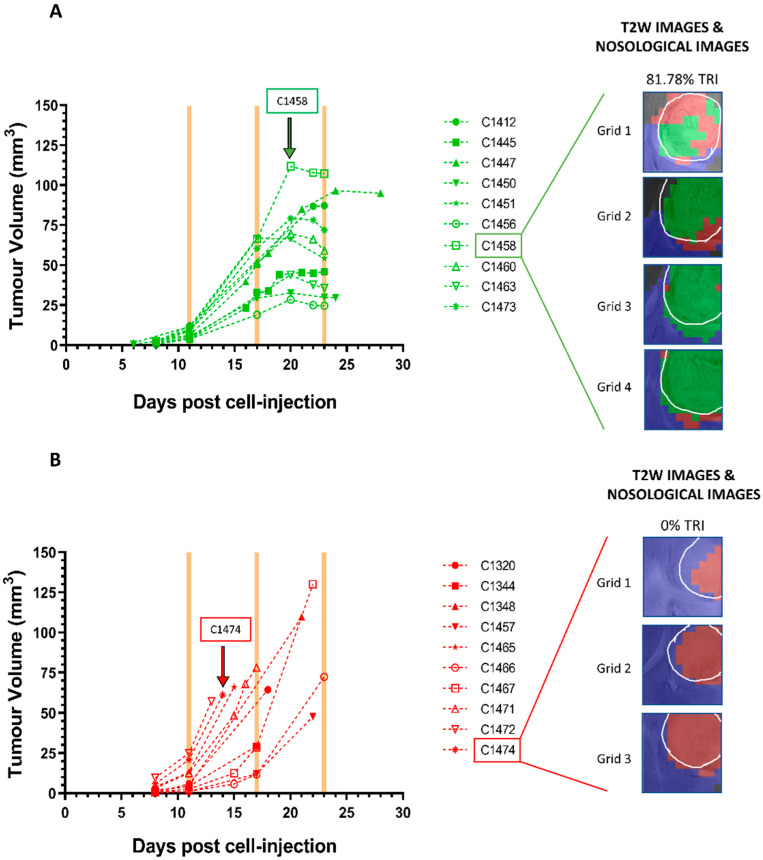
Tumour volume evolution (in mm^3^) of (**A**) responding TMZ-treated (*n* = 10) and (**B**) vehicle-treated (*n* = 10) cases. In all responding cases, tumour volumes were in growth arrest, while in all control cases, tumour volume increased fast. Yellow shaded columns indicate TMZ administration days. The last point in the tumour volume evolution line designates the euthanasia day. The tumour volume at therapy start (day 11 p.i.) was 7.50 ± 3.22 mm^3^ for TMZ-treated mice and 9.07 ± 8.44 mm^3^ for control mice. At the endpoint, the tumour volume was 58.64 ± 26.43 mm^3^ and the TRI was 76.97 ± 11.22% for TMZ-treated mice, while the tumour volume was 71.61 ± 29.18 mm^3^ and TRI was 4.59 ± 6.31% for control mice. C1458 (unique GABRMN mice identifier) is shown as an example of a responding TMZ-treated mouse, and C1474 is shown as an example of a control mouse. Nosological images obtained from Grids 1–4 of case C1458 and Grids 1–3 of case C1474 were superimposed to the T2w-MRI. The tumour areas from the nosological images have been manually drawn over the tumour (shown in white line), and TRI was calculated from it. Rectangles over arrows at left indicate the cases and time points at which the MRSI-derived nosological images shown at right were acquired.

Tumour volume was followed up by MRI, and MRSI acquisitions were carried out in order to measure the extent of response to treatment using the obtained nosological images. Extreme values for TRI were searched for, as well as homogeneous response levels, avoiding heterogeneous samples as much as possible. For TMZ-treated mice, MRSI studies were performed when tumour volume showed decrease in comparison with previous explorations, meeting criteria for “stable disease” according to adapted RECIST [23] (see Supplementary Material file). The time point chosen for TMZ-treated mice was when TRI values were equal or higher than 60%, and average TRI obtained was 76.97 ± 11.22%, corresponding to intermediate/high response categories (see [24] for category definition). The average tumour volume was 58.64 ± 26.43 mm^3^, at day 23.6 ± 1.6 p.i. For vehicle-treated mice, the time point chosen for study was at the moment tumours showed TRI = 0% (or as close as possible, average 4.59 ± 6.31%) and tumour had enough size to provide samples for qPCR experiments (71.61 ± 29.18 mm^3^ at day 18.3 ± 3.8 p.i.). At chosen time points, mice were euthanised by cervical dislocation, brain was removed, and tumour was resected. All collected samples are described in Table S1, and tumour volume evolution is shown in Figure 1.

### 3.2. Microglia/Macrophage Global Population, As Well As M1 and M2 Subtypes, Are Increased in IMS-TMZ-Treated Tumours

In order to characterise the microglia/macrophage population in the GL261 GB microenvironment during TMZ treatment, gene expression level analyses were performed in TMZ-treated and control mice: the F4/80 gene as general GAMs marker, Nos2 gene as M1 subtype marker, and CD206 gene as M2 subtype marker.

**Figure 2:**
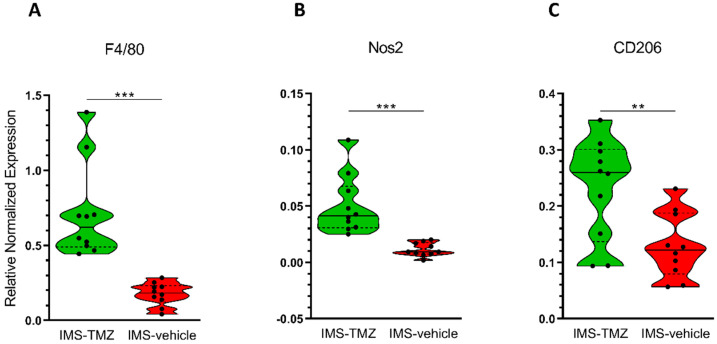
Violin plot for estimation of different cell populations in IMS-TMZ-treated GL261 GB and control mice, for comparisons of (**A**) global GAMs population through F4/80 expression level analysis (*p* = 0.0004), (**B**) M1 macrophage subtype population through Nos2 expression level analysis (*p* = 0.001), and (**C**) M2 macrophage subtype population through CD206 expression level analysis (*p* = 0.009). Data are the mean ± S.D. and significant differences between groups are indicated by asterisks (*** *p* ≤ 0.001, ** *p* < 0.005), *n* = 10 for each group. Solid horizontal line in violin plots indicates median and the dashed lines indicate 1st and 3rd quartiles. Note that graphs are shown with different “y” scaling for better appreciation of data distribution.

F4/80 gene showed significantly higher expression levels in TMZ-treated group than in control group (*p* < 0.0001), with 0.71 ± 0.32 relative expression for treated tumours vs. 0.18 ± 0.08 for control tumours. The same trend was observed for Nos2 gene (0.05 ± 0.03 relative expression for treated tumours vs. 0.01 ± 0.01 for control tumours) and CD206 gene (0.23 ± 0.09 relative expression for treated tumours vs. 0.13 ± 0.06 for control tumours), with significant differences (*p* = 0.0002 and *p* = 0.0073, respectively). Table 1 shows detailed description of gene expression levels and Figure 2 shows gene comparisons. These results suggest that both M1 and M2 subtype populations may increase in TMZ-treated tumours when compared to vehicle-treated tumours, also supported by the larger global microglia/macrophage population detected with the F4/80 gene expression level. 

Moreover, we were also interested in assessing whether the increased levels of global GAMs population would reflect predominant M1 or M2 subtypes. In order to gain more insight into this question, correlation studies were performed with the aforementioned markers F4/80, Nos2 and CD206. Pearson correlation showed significant values for F4/80 vs. Nos2 expression levels in IMS-TMZ-treated samples (*p* = 0.0096), suggesting a positive association (see Figure S2A). This association was not seen in vehicle-treated samples, while CD206 expression was not significantly correlated with F4/80 in any of the instances evaluated (see Figure S2B). These results suggest that the global GAMs population biomarker increase observed in IMS-TMZ-treated tumours (ca. 4-fold) would be mainly related to the increase in the M1 phenotype population and, to a lesser extent, to the increase in the M2 phenotype population.

### 3.3. Assessing Different Macrophage Population Subtypes Regarding Global GAMs Values

**Figure 3:**
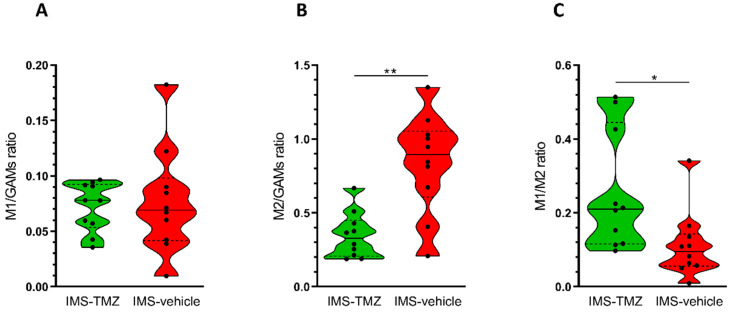
Ratios of (**A**) M1/GAMs, (**B**) M2/GAMs, and (**C**) M1/M2 were calculated in IMS-TMZ-treated (*n* = 10) and control (*n* = 10) tumours using, respectively, the specific markers Nos2 to F4/80, CD206 to F4/80 and Nos2 to CD206. No significant differences were observed (*p* = 0.858) for the ratio of M1/GAMs between TMZ-treated (0.07 ± 0.02 M1/GAMs ratio) and control (0.08 ± 0.05 M1/GAMs ratio) groups. On the other hand, significant differences were found for M2/GAMs ratio (*p* = 0.001) and for M1/M2 ratio (*p* = 0.03) between TMZ-treated (0.35 ± 0.16 M2/GAMs ratio and 0.26 ± 0.16 M1/M2 ratio) and control (0.84 ± 0.34 M2/GAMs ratio and 0.11 ± 0.09 M1/M2 ratio) groups. Data are mean ± SD and significant differences between groups are indicated by asterisks (** *p* < 0.005, * *p* < 0.05). Explanations for violin plots as in Figure 2. Note that graphs are shown in different “y” scaling for better appreciation of data distribution.

Since the purpose of this work was to provide insight about the relationship between microglia/macrophage subpopulations and the MRSI-sampled pattern in control and IMS-TMZ-treated GL261 GB, M1/GAMs and M2/GAMs ratios were analysed (Figure 3A,B) using the corresponding individual markers (F4/80, Nos2, CD206). Thus, in order to check whether the predominant microglia/macrophage subtype population was different in responding IMS-TMZ-treated and vehicle-treated samples, M1/M2 ratio was calculated taking into account the relation of Nos2 gene to CD206 gene expression (Figure 3C). See Table 1 for M1/GAMs, M2/GAMs, and M1/M2 ratio values. Results essentially show that there is indeed a change in the M1/M2 subtype proportions in GL261 tumours upon response to treatment. A significantly higher ratio of M1/M2 microglia/macrophages (*p* = 0.0249) was found in responding IMS-TMZ-treated tumours compared to vehicle-treated tumours, while the ratio M1/GAMs showed no differences (*p* = 0.7944). This apparent discrepancy can be explained when we take into account that both M1 sub type and total GAM increase in responding tumours (Figure 2A,B). Furthermore, lower M2/GAMs ratio (*p* = 0.0006) is also found in responding tumours. Even though M2s are seen to increase in responding tumours (Figure 2C), this increase does not compensate for the higher increase of total GAM, thus their ratio decreases (Figure 3B). 

### 3.4. PD-L1 Gene Is Highly Expressed in IMS-TMZ-Treated Tumours, and These Increases May Be Correlated with the Polarisation State of Microglia/Macrophage Population

**Figure 4:**
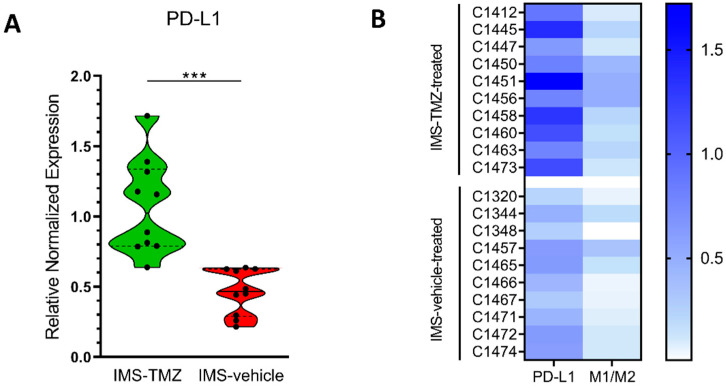
(**A**) Violin plot for comparisons of PD-L1 level analysis expression in tumour samples from IMS-TMZ-treated GL261 GB and control mice (*p* < 0.0001). Data are the mean ± SD and significant differences between groups are indicated by asterisks (*** *p* < 0.001). Explanations for violin plots as in Figure 2. (**B**) Visual coloured map for normalised expression representing PD-L1 expression level (from Figure 4A) and M1/M2 ratio (from Figure 3C) in each individual case. Pearson correlation analysis in the whole group showed overall significance (*p* = 0.013), although when pairs of values were analysed separately for control and IMS-TMZ-treated groups, only the control set of values showed significance (*p* = 0.048), which was lost for values from treated cases.

The PD-L1 gene level expression was assessed, and values were compared between IMS-TMZ-treated and vehicle-treated groups. Results suggest a significantly higher PD-L1 gene expression in responding TMZ-treated tumours in comparison with control tumours (*p* < 0.0001), with a 1.07 ± 0.34 relative expression found for TMZ-treated tumours and a 0.46 ± 0.16 relative expression for control tumours (see Figure 4A for visual comparison). Furthermore, to investigate whether PD-L1 gene expression levels were correlated with the polarisation state of GAMs, Pearson correlation analyses were performed, and PD-L1 expression level was shown to be positively correlated to the M1/M2 ratio (*p* = 0.0127) (Figure 4B and Figure S2C), suggesting that for higher M1/M2 ratios, a higher PD-L1 expression level was found.

## 4. Discussion

### 4.1. Immune System Populations Change during Response to Therapy

During this work, samples from GL261 GB-bearing mice treated with IMS-TMZ (*n* = 10) or vehicle (*n* = 10) were analysed by qPCR in order to characterise the microglia/macrophage population into the tumour site. Tumours were excised at chosen time points, guided by the response level spotted by MRSI-based nosological images, reflecting specific changes in their spectral pattern. Our results reinforce the idea of the microglia/macrophages role in tumour evolution: both control and treated tumours presented relevant microglia/macrophage content. Still, an overall increase (ca. 4-fold change in average) in such content was observed in IMS-TMZ-treated tumours responding to therapy, through F4/80 gene level expression (Figure 2A). This trend is in line with previous Iba-1 immunohistochemistry data [31], which reported a 2.4-fold increase in the percentage of Iba-1 stained area for TMZ-treated tumours (when narrowing groups to control vs. high response cases, see [24,31] for further details). Since different methodological approaches were used in [24,31] and in this work, direct comparison of results is not straightforward, as already described by others [42], although the trend is clear.

Increased infiltration of immune cells into tumour sites after therapy and its relationship with effective response has been described for cancer types such as colorectal [43], breast [44], ovarian [45], and brain [46], in agreement with results described in this work. Immune infiltration has been described as a good prognostic factor, and certain chemotherapeutic agents were described to actually enhance the host immune response through presentation of tumour antigen peptides to T-cells, or upregulation of tumour antigens, rendering these tumour cells more susceptible to immune system attack [47]. This is also related to the corollary that effective therapeutic strategies should convert a ‘cold’ tumour (noninflamed) with low immune cell infiltration into a ‘hot’ tumour (inflamed) with high immune cell infiltration [48].

In our study, response to IMS-TMZ treatment increased the infiltrating microglia/macrophage population. The beneficial effect of TMZ in preclinical/clinical settings was mainly attributed to its effect as DNA alkylating agent and activation of the apoptotic cascade [49,50]. However, it is worth noting that TMZ alone has a cytostatic rather than cytotoxic mechanism, when added to GL261 cultured cells at concentrations similar to the ones used in preclinical studies, as described in [9,51,52,53]. Thus, the main beneficial effects observed with TMZ therapy may have a different explanation. In this respect, the potential immunogenic effects of TMZ are gaining prominence [9,54,55,56,57,58]. Through the release/exposure of immunogenic signals, TMZ administration may launch the cancer immune cycle as described by Chen and Mellman [59], eventually leading to tumour cell killing. On the other hand, it was also described that DNA damage repair (DDR) mechanisms triggered after TMZ exposure could lead to reprogramming of macrophages to a tumor-supportive state (M2) and TMZ-resistant cells would display upregulated DDR cytokines (preclinical and database human samples) [60], in line with overall results described in [61] with GL261 cells. Hence, TMZ can have an indirect role in microglia/macrophage polarisation changes, with an M2 polarization being supported by different authors [62,63], although none of them used an IMS schedule. Still, we should also have in mind a possible indirect effect of TMZ over the lymphocytes anergy status after striking the tumour, which may superimpose in time with the periodic TMZ administration. One of the goals of our work was to investigate whether local changes in IMS-TMZ-treated tumours, especially with respect to immune system, would be related to MRSI-detected spectral pattern changes, as previously proposed [24,25,31]. The cancer immune cycle involves several cellular populations and local factors. However, in order to be detected by MRSI-based approaches (i.e., to significantly contribute to the spectral pattern), these cellular populations must represent a significant proportion of the tumour volume. Hence, the GAM population is a suitable candidate to be at least partially responsible for the observed changes, since they are the most common non-tumoural cell within GB masses, reaching values up to 30–40% of the overall volume [5]. Other cellular populations such as lymphocytes would represent only a small fraction of the GB mass volume (ca 1% in GL261 GB, unpublished GABRMN data) and may not have a direct impact in spectral pattern changes. 

### 4.2. Not All GAMs Are Equal (I): Polarisation of Microglia/Macrophages and Its Role in Therapy Response in GB

In the majority of solid tumours, progression is associated with a phenotype prevalence changing from M1 to M2 [64], which can trigger the suppression of effector T cell immunity, improved tumour cell survival, promotion of angiogenesis, and chemoresistance [65]. In this sense, we assessed the GAM polarisation profile when GL261 tumours showed a clear MRSI-detected responding pattern and compared them with untreated control tumours. Our goal was to better understand the oscillatory behaviour of the MRSI-detected pattern in terms of a transient/permanent response to therapy in GL261 GB [24,31]. 

Our studies with Nos2 and CD206 gene markers suggest, first, that both control and IMS-TMZ-treated tumours contain M1 and M2 microglia/macrophages (Figure 3A,B), in line with data for control GL261 tumours described in [66]. More interestingly, our results point to a significantly higher ratio of M1/M2 microglia/macrophage content in IMS-TMZ-treated responding tumours (Figure 3C), being ca. 2.3-fold times higher than in control tumours. This result indicates a phenotype switch of GAM from M2 to M1 in responding tumours compared to control tumours. In other words, more cells infiltrating the tumour with the ability to start and sustain inflammatory responses and to exhibit antitumour activity and leading to tumour tissue disruption. This specific M1/M2 ratio was also evaluated by other authors in various cancer tumour types and in relation to outcome prognosis. For example, high content of M1 macrophages was associated with best prognosis in treated ovarian cancer patients, suggesting a correlation between efficacy of antineoplastic regimens and M1 polarisation [67]. On the other hand, authors in [68] studied the prevalence of M2-polarised macrophages in different lung neoplastic lesions, with high M2 infiltration predicting poor prognosis. In lung adenocarcinomas, 79.71% of tumour-associated macrophages were M2 polarised and the remaining 20.35% were M1 polarised [68]. Regarding brain tumour-related studies, investigation with clinical glioma samples have found an association between high CD163^+^ cells (M2 marker) and glioma progression [69]. In the same line, authors in [37] have found that predominant M1 polarisation was associated with better overall prognosis of GB patients, using CXCL10 and CCL13 as markers for M1 and M2 macrophages, respectively. Moreover, the M1/M2 ratio was described to be correlated with survival rate in GB patients after TMZ treatment [70]. Further work reported in [71] revealed mixed profiles of M1 and M2 macrophages in human GB while the ratio M1/M2 correlated with survival in IDH1 wild type GB. It is then clear that achieving suitable M1/M2 ratios is desirable and will be determinant for outcome in preclinical and clinical GB. 

### 4.3. Not All GAMs Are Equal (II): The Polarisation Status of Microglia/Macrophages Affects Their Metabolomic Pattern

It has been described that microglia/macrophage polarisation implies metabolic changes, involving pathways such as glycolysis, the Krebs cycle, and fatty acid metabolism, enabling the specialised activities of these cells [72,73]. Furthermore, changes in key metabolic regulatory events in microglia/macrophages can be initiated in response to changes in the tumour microenvironment [74]. In relation to this, M1 macrophages rely on glycolysis for energy production, while M2 macrophages mostly use mitochondrial oxidative metabolism (Krebs cycle) for ATP biogenesis. This entails that M1 microglia/macrophages increase lactate release and fatty acid synthesis, while the synthesis of N-acetyl group-containing compounds, glutamine, glycine, and alanine, among others, is upregulated in the M2 microglia/macrophage population [11,72,75,76]. Besides, the differential induction of fatty acid synthesis and fatty acid oxidation elicits microglia/macrophage polarisation towards M1 or M2 profiles, respectively [11]. 

The different metabolic pathways should contribute, at least partially, to the differential metabolomic pattern detected in vivo by MRSI-based analyses in GL261 GB transiently responding to therapy compared with control, untreated tumours. In this respect, the prototypical source of TMZ-responding tumours [23] shows large amounts of mobile lipids/lactate (1.33 ppm signal) and increased lactate at 4.1 ppm, combined with higher polyunsaturated fatty acids (PUFA, 2.8 ppm signal) and other changes in the glutamine, glutamate, and alanine regions, which would be consistent with the metabolic profiles expected for the M1 subtype compared to the M2 subtype (e.g., higher lactate in M1s), related to the limitation of pyruvate entrance into the Krebs cycle triggering formation of high amounts of lactate from pyruvate [77]). 

However, more detailed studies will be needed to further assess the actual impact of metabolic changes related to microglia/macrophage polarization on the in vivo MRSI differential metabolomic pattern. In this respect, it is worth remembering that the tumour spectroscopic pattern also contains contributions from tumour cells and extracellular metabolites/macromolecules/lipids present in the tumour microenvironment in suitable concentration to be detected by MRSI. In any case, the MRSI-based nosological images and the changes in spectral pattern (sources) behind those images seem to be coherent with changes in M1/M2 ratios. This reinforces the potential of those images for early in vivo detection of whether a certain therapeutic approach is properly eliciting efficient host immune response against GB tumours. 

Finally, we may also consider that literature describes M2 macrophages being able to re-polarise to M1, but not the reverse; M1 macrophages are mostly consumed during the inflammatory response [78]. Then, after a first “wave” of macrophage M1 polarisation and tumour cell killing, probably driven by interaction with T-cells [9], M1 sub-population decrease would bring down M1/M2 ratios, close to levels found in untreated actively proliferating tumours. Figure S3 combines a hypothetic scheme of events taking place during IMS-TMZ therapy in GL261 GB demonstrated in this work (GAM changes, M1/M2 polarisation) with the corresponding changes in the MRSI-based nosological images described in previous work [23,24,25,31] and used here to select adequate time points for sampling. Its potential relevance for translational studies is also highlighted there. 

### 4.4. PD-L1 Gene Expression in GL261 GB

PD-L1 expression level is considered a major positive prognostic biomarker for immune therapy in many cancers, but not yet in glioma [79]. The expression and subcellular distribution of PD-L1 in the tumour tissue exhibits great variability reflecting the specificities of cellular and structural microenvironment in the brain, preventing the confident use of PD-L1 as a prognostic biomarker in glioma [80].

Results obtained in the present work have assessed the PD-L1 gene level expression in IMS-TMZ-treated and vehicle-treated GL261 GB groups. A significantly higher PD-L1 gene expression (2.3-fold) was found in responding TMZ-treated tumours compared to untreated ones. Chemotherapy has been described to modify the tumour microenvironment, increasing PD-L1 expression [81,82], and our previous work with Western-blot analysis for control, treated-relapsing, and unresponsive data from GL261 GB samples [31] seems to partially agree with those results. This is in line with results described by authors in [83], who also suggested that PD-L1 increase was related to STAT3 signalling. Furthermore, a significant positive correlation was found between PD-L1 gene expression level and the M1/M2 ratio (see Figure 4B and Figure S2C), indicating that increased PD-L1 content could be associated with increased M1-polarised macrophages.

PD-L1 is known to be expressed in a variety of cell types, including macrophages. For example, a study of patient GB samples showed that monocytes cultured in glioma-conditioned medium expressed high levels of PD-L1 [84]. The role of PD-L1 in tumour cells and macrophages seems to be different, though. An interesting study with murine melanoma and breast cancer cells by Singhal et al. [85] showed that only PD-L1 on target tumour cells clearly inhibited the effector functions of T cells. PD-L1 expressed by macrophages exerted a regulatory role only during the interaction of macrophages presenting tumour antigen to effector T cells. Thus, in this case PD-L1 could be simply limiting excessive activation of T cells and protecting PD-L1 harbouring macrophages from being killed by approaching T cells [85]. In short, available evidence indicates that PD-L1 from both tumour cells and microglia/macrophages is relevant for assessing prognosis [86]. In our case, the origin of the reported PD-L1 expression level changes is still unclear, since samples analysed contain both tumour cells and GAMs and further studies will be needed for better clarification of this extreme. 

### 4.5. Wrap-up: Incoporating the Measured Gene Expression Results into the Explanation of the Oscillatory Behaviour of the MRSI-Based Biomarker of Response

In longitudinal studies of IMS-treated response assessment, we have reported periodic oscillations of the MRSI-based biomarker showing increases in the detected response level (TRI) every 6 days. In this sense, we also assume that there are tissue events/changes that originate this oscillation, alternating a high response nosological image profile with periods showing tissue characteristics that resemble more an actively proliferating tumour. 

Figure S3 summarises how we correlate cellular/molecular events taking place with results obtained in this work. At the therapy starting point, GL261 GB tumours display an essentially protumoural microglia/macrophage phenotype, supported by qPCR results and encoded in the red colour over the tumour mass of the nosological images. TMZ therapy may lead to release/exposure of immunogenic signals [53], which set the cancer immunity cycle [59]. In the meanwhile, both M1 and M2 populations increase (see also Figure 2A): M1 microglia/macrophages will participate in tumour cell killing [9], and M2 microglia/macrophages are waiting for M1 polarisation. The M1/M2 ratio changes towards higher values in tumours showing transient response to IMS-TMZ (Figure 3C). Maximum response is spotted by our noninvasive biomarker (green colour in nosological images) after ca. 6 days of therapy administration, in line with the length of the immune cycle described in [32]. At this point, an increase in PD-L1 gene expression is observed (Figure 4A), although its origin could be either in tumour cell population, microglia/macrophages or both together. Since M1 microglia/macrophages are mostly consumed during the antitumour response events, after such interval, the ratio M1/M2 may shift towards the control values, T lymphocytes may be approaching exhaustion, and surviving tumour cells may start proliferating again leading to tumour regrowth (day +9, red colour over the tumour image) until the previous therapeutic administration point (at day +6) resets the immune cycle and produces the next response oscillation (day +12).

Having this hypothesis in mind, how can we obtain an advantage regarding this biomarker? It is worth noting that therapy can modulate/change macrophage profiles towards an inflammatory M1 profile with satisfactory results, as already described in preclinical GB models using amphotericin B [87], CSF-1R inhibition [88], immunovirotherapy [89], or recombinant adeno-associated virus [90], although the latter presented only modest result in phase II clinical phases [91]. On the other hand, work described in [92] focused into blocking relevant pathways in protumoural macrophages with minocycline in preclinical murine GB models. Our results suggest that our imaging biomarker findings are at least partially explained by changes in microglia/macrophage profiles within brain tumours (hence, in the expected antitumour/protumour actions). Thus, we may be able, in a near future, to follow-up different therapies and foresee results in an early fashion gathering hints about the prevailing macrophage population at a given moment. A word of caution may be issued here, since the mutational load of GL261 [93] is much higher than untreated human GB [94] and this could be a determinant for host immune system eliciting. In this respect, relapsing human GB contain a similar mutational load [95] to GL261 tumours. Further confirmation of the potential of such imaging biomarker in human GB therapy response follow-up may be worthwhile to investigate.

## 5. Conclusions

Our results confirm that TMZ administered in an immune-enhancing metronomic schedule increases the GB-associated microglia/macrophage population infiltrating the tumour. The M2/GAMs ratio was shown to be remarkably lower in responding IMS-TMZ-treated mice, while the M1/M2 ratio was significantly higher when compared to vehicle-treated mice. These results indicate that TMZ treatment applied in IMS protocols contributes to immune system activation, suggesting M2-to-M1 polarisation, improving the anti-tumoural response mediated by microglia/macrophages. Since it is well known that GAMs can represent 30–40% of cells in GB and M1 and M2 microglia/macrophages have different metabolic profiles, this relative population change could be one of the reasons for the differential MRSI-sampled pattern during response to therapy reinforcing its proposed role as immune system activity biomarker for future work.

It remains unclear whether the increase of the PD-L1 gene level expression in the responding IMS-TMZ-treated tumours originates from changes in tumour cells, the M1/M2 microglia/macrophages polarisation, or both. Further studies will be needed to assess the relative roles of the two cell types in the detected increase of the PD-L1 expression upon response to therapy in the GL261 GB.

## Figures and Tables

**Table 1:** Relative normalised expressions obtained in qPCR studies with IMS-TMZ and IMS-vehicle-treated samples: average ± SD of F4/80, Nos2, CD206, and PD-L1 genes, and ratios of M1/GAMs, M2/GAMs, and M1/M2. Significant differences between groups are indicated by asterisks (*** *p* ≤ 0.001, ** *p* < 0.005, and * p < 0.05)).

	Relative Expression						
	F4/80 ***	Nos2 ***	CD206 **	PD-L1 ***	M1/GAMs	M2/GAMs **	M1/M2 *
IMS-TMZ-treated	0.71 ± 0.32	0.05 ± 0.03	0.23 ± 0.09	1.07 ± 0.34	0.07 ± 0.02	0.35 ± 0.16	0.26 ± 0.16
IMS-vehicle treated	0.18 ± 0.08	0.01 ± 0.01	0.13 ± 0.06	0.46 ± 0.16	0.08 ± 0.05	0.84 ± 0.34	0.11 ± 0.09

## Data Availability

The data presented in this study are available on request from the corresponding author.

## Supplementary Materials

The following are available online at https://www.mdpi.com/article/10.3390/cancers13112663/s1, Table S1: Description of IMS-TMZ and IMS-vehicle treated mice, including tumour volume at therapy start point and at endpoint, euthanasia day and percentage of TRI shown at that time; Figure S1: Summary of steps performed for nosological images calculation mentioned in this work; Figure S2: Pearson correlation analysis between F4/80 gene expression level and (**A**) Nos2 and (**B**) CD206 gene expression levels; and between (**C**) PD-L1 gene expression level and M1/M2 ratio (see section 3.4. of the main manuscript for definition) in IMS-TMZ-treated GL261 GB (green line) and control (red line) mice samples. (**C**) Also shows the correlation between PD-L1 gene expression level and M1/M2 ratio when treated and control cases are combined (black line). P values for each group are indicated in the graphs; Figure S3: Hypothetical scheme for the rationale behind changes in the nosological images coding for response in MRSI of IMS-TMZ treated GB GL261 bearing mice (see main text for further details).
