# Supplementary material for: Immune System-Related Changes in Preclinical GL261 Glioblastoma under TMZ Treatment: Explaining MRSI-Based Nosological Imaging Findings with RT-PCR Analyses
Source: Cancers (Basel). 2021 May 28;13(11):2663. doi: 10.3390/cancers13112663 (PMC8199490; doi:10.3390/cancers13112663)
Supplement: Supplementary file 1 [file cancers-13-02663-s001.zip › cancers-1169241-supplementary -revised-final-2.pdf]

cancers

MDPI

# Supplementary Materials: Immune System-Related Changes in Preclinical GL261 Glioblastoma under TMZ Treatment: Explaining MRSI-Based Nosological Imaging Findings with RT-PCR Analyses

**Pilar Calero-Pérez, Shuang Wu, Carles Arús and Ana Paula Candiota.**

## Supplementary Introduction

*MRSI analysis: outline of NMF methods*

Non-negative matrix factorization (NMF) methods are multivariate data analyses designed for meaningful components (also known as sources) estimation, originating from non-negative data. Standard NMF methods decompose data "X" into 2 non-negative matrices: sources ("S") and mixing matrices ("A"). The divergence between X and S*A is measured by cost functions, which may differ for different NMF methods. There are NMF variants which can also handle negative data, such as convex-NMF, which was the basis of the analysis used in this study [1]. Convex-NMF was capable to spot a reduced number of sources confidently recognized as representative from brain tumour/tissue types much better than other NMF variants [2,3]. Original sources were extracted from TMZ-treated and control mice individuals, as described in [4], defining prototypic metabolomic patterns related to normal or unaffected brain, GL261 tumours actively proliferating and TMZ-treated, responding tumours. The source-based analysis used in this work and other studies mentioned [5–7] made use of previously extracted sources in order to study new cases and assign one of the three predefined classes to each investigated voxel, thus generating the so-called nosological images.

## Supplementary Materials and Methods

*MRI studies*

The acquisition parameters for MRI studies were as follows: repetition time (TR)/effective echo time (TEeff) = 4200/36 ms; echo train length (ETL) = 8; field of view (FOV) = 19.2 × 19.2 mm; matrix size (MTX) = 256 × 256 (75 × 75 µm/pixel); slice thickness (ST) = 0.5 mm; inter-ST = 0.1 mm; number of slices (NS) = 10; number of averages (NA) = 4; total acquisition time (TAT) = 6 min and 43 s.

*MRSI studies*

Grid 1 was the first upper (dorsal) grid and was acquired with 10 × 10 matrix size. Then, Grid 2 was acquired 1 mm below using 12 × 12 matrix size, and grid 3 was acquired 1 mm below Grid 2, with the same matrix size. Finally, in case that tumour volume was not completely covered, a final Grid 4 was acquired 1 mm below Grid 3 with 10 × 10 matrix size.

Variable Power and Optimized Relaxation Delay (VAPOR) was used for water suppression, using a 300-Hz bandwidth. Fast Automatic Shimming Technique by Mapping Along Projections (FASTMAP) was used for linear and second order shims automatic adjustment. Finally, 6 saturation slices (ST, 10 mm; sech-shaped pulses: 1.0 ms/20250 Hz) were positioned around the VOI for minimizing outer volume contamination in the signals obtained.

Acquisition parameters for all grids were as follows: FOV, 17.6 mm × 17.6 mm; VOI in Grids 1 and 4 was 5.5 mm × 5.5 mm × 1.0 mm, while Grids 2 and 3 had 6.6 mm × 6.6 mm × 1.0 mm. TR, 2500 ms; ST, 1 mm; Sweep Width (SW), 4006.41 Hz; NA, 512; TAT, 21

min 30 s each grid. Water suppression was performed with Variable Power and Optimized Relaxation Delay (VAPOR), using a 300 Hz bandwidth. Linear and second order shims were automatically adjusted with Fast Automatic Shimming Technique by Mapping Along Projections (FASTMAP) in a 5.8 mm × 5.8 mm × 5.8 mm volume which contained the original VOI region. Six saturation slices (ST, 10 mm; sech-shaped pulses: 1.0 ms/20250 Hz) were positioned around the VOI to minimize outer volume contamination in the signals obtained.

*Nosological images*

The nosological images are generated combining MRSI techniques with robust machine learning analyses, which take the whole spectral pattern changes into account (considers the 0 to 4.5 ppm spectral vector, see figure S1B). This makes it possible to use quantitative changes of different metabolites and to detect even subtle variations in metabolomic profile, enabling to classify the tissue into mostly (majority source vote) normal brain tissue, responding or unresponsive tumour.

The labelling of the MRSI data acquired is based on the source extraction technique, which assumes that a mixture of the heterogeneous tissue patterns is present in each voxel and that the contribution of individual source to the final pattern can be calculated. In this sense, the nosological imaging is generated by estimating the contribution of each source ("paradigmatic spectra") to the individual voxels in the MRSI grid in order to assign each acquired voxel to one of the predetermined classes. The quantitative most relevant paradigmatic spectrum of the voxel is selected as the "winning source" and the voxel is correspondingly coloured, finally represented as nosological maps for each matrix. Green colour is used when the GB responding to treatment source contributes the most, red for actively proliferating GB, blue for normal brain parenchyma, and black for undetermined tissue. These nosological images can provide a visual representation of MRSI results and be used as an imaging biomarker to determine therapy-caused response.

The most remarkable changes between responding and unresponsive tumours can be summarised. Main metabolites contributing to different patterns of responding and non-responding spectra are mobile lipids 0.9 + macromolecules (ML, MM, 0.9 ppm), mobile lipids 1.3 + lactate (ML/Lac, 1.3 ppm), N-acetyl-aspartate and N-acetyl group-containing compounds (NAA and NAc, 2.02 ppm), glutamate + glutamine (Glx, 2.1–2.4 ppm), polyunsaturated fatty acids in mobile lipids (PUFA, 2.8 ppm), total creatine (Cre, 3.03 ppm), choline-containing compounds (Cho, 3.21 ppm), myo-inositol + glycine (Ins + Gly, 3.55 ppm), glutamine + glutamate (Glx, 3.8 ppm, which is also partially contributed by alanine), and lactate (Lac, 1.3 and 4.1 ppm).

Figure S1 provides a summary of the steps performed in this type of analysis.

*Inclusion criteria for mice fully evaluated in this study*

Not all studied mice accomplished criteria to be included in this particular study, which searched for homogeneous and clear control and therapy-responding examples to explain the associated MRSI patterns. Inclusion criteria were as follows:

1. Homogeneous tumour appearance at MRI exploration, avoiding tumour volumes at inclusion decision time outside the usual tumour volume range values (average 9.3 ± 6.1 $mm^3$) considered in current GABRMN group cohort studies [5–9]. Tumours growing towards the skull or lower part of the brain, in which MRSI signals can be spurious and have poor spectral quality, were also discarded. Total number discarded due to criteria 1 = 44.
2. Extreme values for Tumour Responding Index (See [5]) were searched for (i.e., TRI values >60% for treated tumours and close to 0% for control tumours). In addition, not only the TRI value itself but its homogeneous distribution within the tumour mass

was preferred, since it was not feasible to 'dissect' samples in different tumour regions. Thus, tumours showing heterogeneous response pattern distributions in nosological images were avoided. Total number discarded due to criteria 2 = 30.

3. Maintenance or reduction in the tumour size was searched for in the treated group, pointing towards clear growth arrest. The reduction was mostly in agreement with 'stable disease' according to RECIST values (4.43 ± 6.32% tumour volume reduction in comparison with previous explorations). The lack of further follow-up prevents us to define whether it would meet partial or complete response criteria. Total number discarded due to criteria 3 = 3.
4. Tumour tissue available volume had to be large enough (more than 20 $mm^3$) to obtain the amount of RNA suitable to carry out qPCR experiments (i.e. > 70 ng/μL). Total number discarded due to criteria 4 = 13.

It is worth noting that mice discarded from this study because of inclusion criteria, were allocated to other studies performed by our group in order to minimize animal waste and maximize knowledge obtained from this particular animal model.

*Adapted RECIST criteria*

Classification based on adapted RECIST criteria was applied as follows, considering the GL261 GB tumour volumes registered in the previous exploration:

- Progressive disease (PD): 20% increase in tumour volume, or higher.
- Partial response (PRe): tumour volume decreases by 30%, or higher.
- Stable disease (SDi): less than 20% increase and no more than 30% decrease in tumour volume.

*Amplicon Context Sequence*

- F4/80:
TATGCCATCCACTTCCAAGATGGGTTAACATCCTTTCTTGCTTTTAAATATATTATGGAACAATGTCTGAAGATTGTAAGTG
CTTTAAGGATCATACTTTTAATAAC
- Nos2:
ACCACACCAAACTGTGTGCCTGGAGGTTCTGGATGAGAGCGGCAGCTACTGGGTCAAAGACAAGAGGCTGCCCCCCTGCT
CACTCAGCCAAGCCCTCACCTACTTCCTGGACATTACGACCCCTCCCACCC
- CD206:
TGGAATAATATCCACTGTTCTTCGTACAAAGGATTTATTTGTAAAATGCCAAAAATTATTGATCCTGTAACTACACACTCAT
CCATTACAACCAAAGCTGACCAAAGGAAGATGGATC
- PD-L1:
GCTAGATGTGGAGAAATGTGGCGTTGAAGATACAAGCTCAAAAAACCGAAATGATACACAATTCGAGGAGACGTAAGCA
GTGTTGAACCCTC
- TBP:
TCTGAGTACTGAAGAAAGGGAGAATCATGGACCAGAACAACAGCCTTCCACCTTATGCTCAGGGCTTGGCCTCCCCACAG
GGCGCCATGACTCCTGGAATTCCCATCTTTAGTCCAATGATGCCTTACGGCA
- HPRT:
TTTTATCAGACTGAAGAGCTACTGTAATGATCAGTCAACGGGGGACATAAAAGTTATTGGTGGAGATGATCTCTCAACTTT
AACTGGAAAGAATGTCTTGA

**Supplementary Figures:**

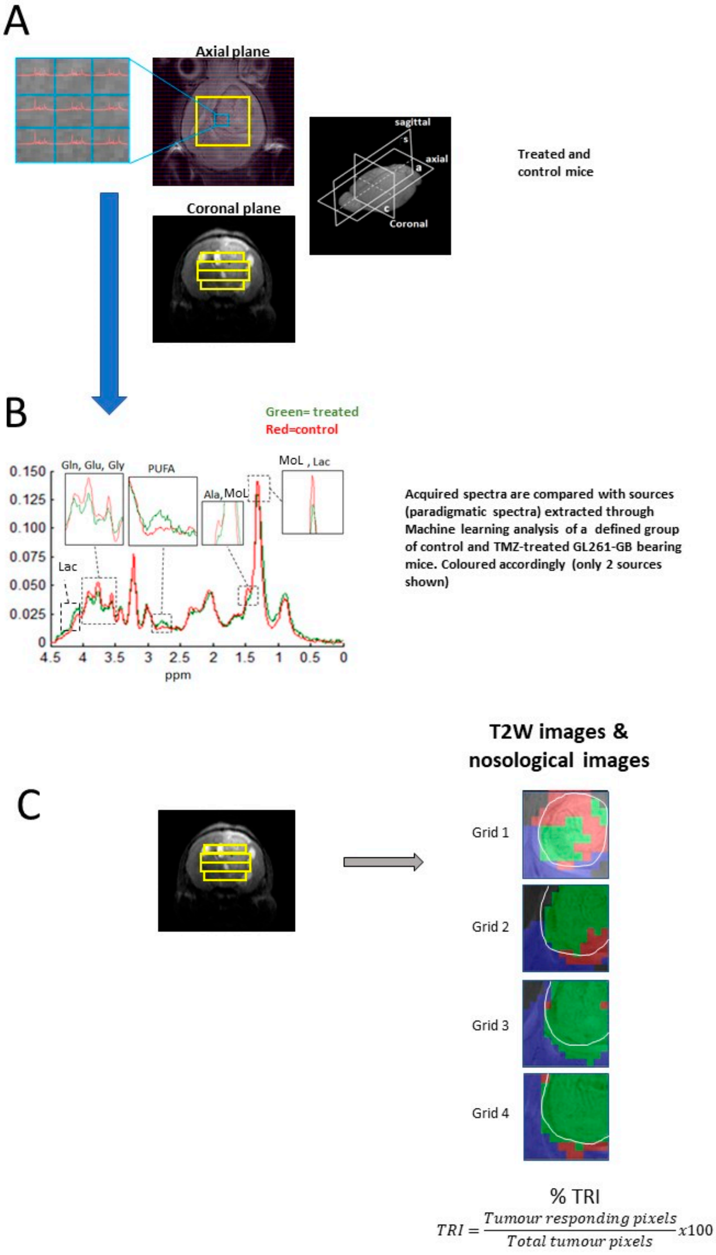


**Figure S1.** Summary of steps performed for nosological images calculation mentioned in this work. **A**) MRSI spectra are acquired. Both coronal and axial imaging orientation are shown for better understanding of different anatomical levels studied. Each individual spectrum of the MRSI grid (blue square in A) is analysed as a unique spectral vector. **B**) Spectra are compared with fixed sources (paradigmatic spectra), obtained from a defined group of TMZ-treated and control mice [4]. In fact, 3 sources were acquired but only sources corresponding to control – actively proliferating- and TMZ-treated mice, transiently responding to therapy, are shown, respectively in red and green. Those are the sources relevant to this particular study. Main contributing metabolites are shown in expansion regions. **C**) Spectra, and volume elements, are then coloured according to whether they show more correlation with the control, TMZ-treated or normal brain parenchyma source. If the correlation between the spectrum of a voxel and the sum of the percentages contributed by the sum of the three tissue sources was below a threshold of 50%, this voxel was labelled as ‘undecided’ and coloured in black [4]. The Tumour Responding Index (TRI) is then calculated as shown in the formula at the bottom, considering spectra (pixels) identified as responding regarding to the total tumour pixels, taking into account manual drawing over the abnormal section in T2w MRI.

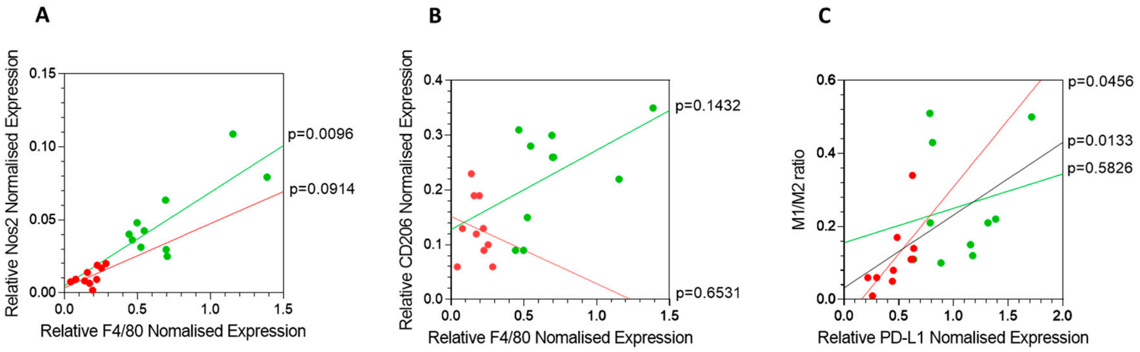


**Figure S2.** Pearson correlation analysis between F4/80 gene expression level and **A**) Nos2 and **B**) CD206 gene expression levels; and between **C**) PD-L1 gene expression level and M1/M2 ratio (see section 3.4. of the main manuscript for definition) in IMS-TMZ-treated GL261 GB (green line) and control (red line) mice samples. **C**) Also shows the correlation between PD-L1 gene expression level and M1/M2 ratio when treated and control cases are combined (black line). P values for each group are indicated in the graphs.

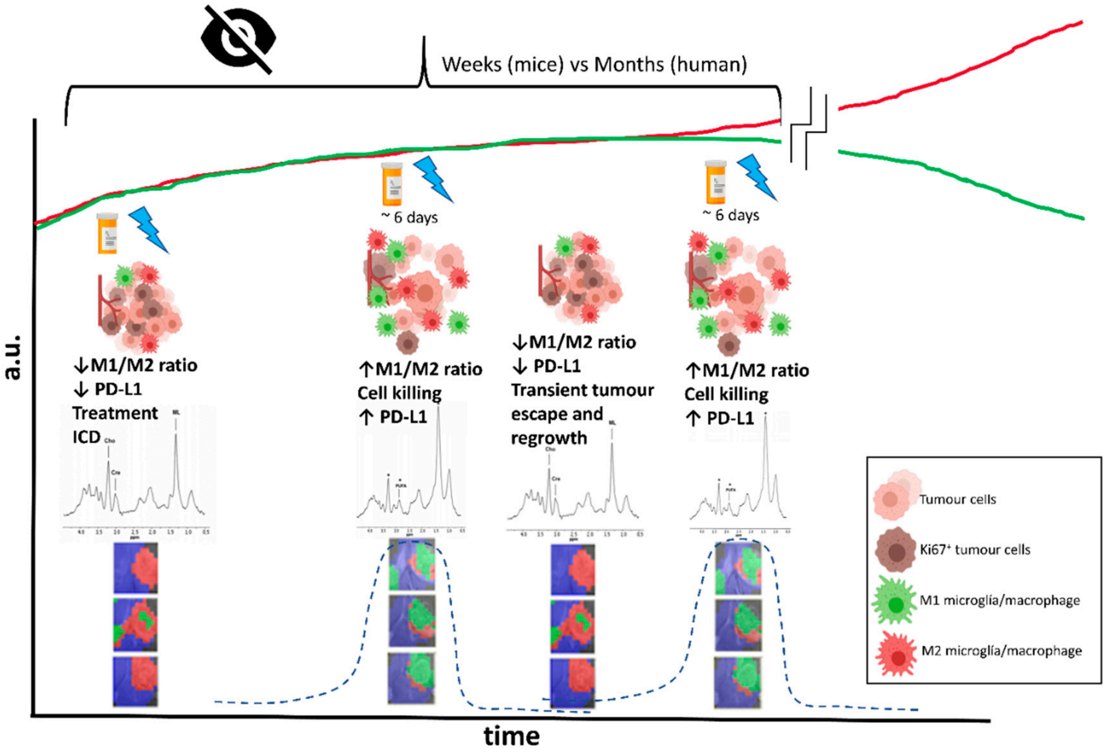


**Figure S3.** Hypothetical scheme for the rationale behind changes in the nosological images coding for response in MRSI of IMS-TMZ treated GB GL261 bearing mice (see main text for further details). The oscillation in cellular populations such as Ki67 positive tumour cells or microglia/macrophages, changes in their polarisation status, in surface receptor such as PD-L1 and their subsequent effects, as well as changes in tissue microstructure (giant cells, acellular spaces [5,10]) should all contribute to the MRSI-detected imaging biomarker differences. Those show periodic oscillations that agree with the length of the immune cycle (ca. 6 days) and should be related to events triggered by TMZ therapy.

This may be extended [7] to any therapeutic approach eliciting the host immune system. It is also worth noting that these events take place early in the timeline, far before any changes are seen in tumour volumes (red and green lines). Hence detection of such oscillatory changes could provide early indication about immune system-driven response to therapy, while tumour volume changes may be 'blind' to these early local metabolomics changes. At the therapy starting point, GL261 GB tumours display an M2/GAMs ratio which is more than 10-fold higher the M1/GAMs ratio, thus an essentially protumoural microglia/macrophage phenotype (encoded in the red colour over the tumour mass of the

nosological images). Launching TMZ therapy may trigger immunogenic cell damage and release/exposure of immunogenic signals [11] which will turn on the cancer immunity cycle and elicit the host immune system [12]. In the meanwhile, both M1 and M2 populations infiltrating the tumour increase (see also Figure 2A): while M1 microglia/macrophages will participate in tumour cell killing, probably after interaction with T-cells [13], and M2 microglia/macrophages are waiting for M1 polarisation. In this respect, M1/M2 ratio changes towards higher values in tumours showing transient response to IMS-TMZ (Figure 3C of the main manuscript). The point of maximum response spotted by our non-invasive biomarker (i.e., green colour over the GB tumour mass in nosological images) is ca. 6 days after therapy administration, in line with the length of the immune cycle described in [14]. At this point, an increase in PD-L1 gene expression is observed (Figure 4A of the main manuscript), although it could be either due to expression by the tumour cell population to evade lymphocyte attack, to expression in microglia/macrophages or both together. Since M1 microglia/macrophages are mostly consumed during the antitumour response events, after such interval, the ratio M1/M2 may shift towards the control values, T lymphocytes may be approaching exhaustion and surviving tumour cells may start proliferating again leading to tumour regrowth (day +9, red colour over the tumour image) until the previous therapeutic administration point (at day +6) resets the immune cycle and produces the next response oscillation (day +12).

## Supplementary Tables:

**Table S1.** Description of IMS-TMZ and IMS-vehicle treated mice, including tumour volume at therapy start point and at endpoint, euthanasia day and percentage of TRI shown at that time.

| Group | Case | Tumour volume at therapy start point ($mm^3$) | Tumour volume at endpoint ($mm^3$) | Euthanization (day p.i.) | TRI (%) |
|---|---|---|---|---|---|
| IMS-TMZ | C1412 | 12.05 | 87.17 | 23 | 95.21 |
| | C1445 | 3.74 | 45.99 | 23 | 60.13 |
| | C1447 | 6.17 | 94.92 | 28 | 78.70 |
| | C1450 | 5.45 | 29.61 | 24 | 72.83 |
| | C1451 | 11.60 | 54.09 | 23 | 64.24 |
| | C1456 | 3.63 | 24.50 | 23 | 71.18 |
| | C1458 | 9.95 | 107.24 | 23 | 81.78 |
| | C1460 | 9.30 | 59.12 | 23 | 93.57 |
| | C1463 | 4.37 | 35.55 | 23 | 76.47 |
| | C1473 | 8.69 | 71.96 | 23 | 75.61 |
| IMS-vehicle | C1320 | 5.80 | 64.36 | 18 | 3.26 |
| | C1344 | 4.26 | 29.30 | 17 | 0.00 |
| | C1348 | 4.35 | 109.67 | 21 | 5.37 |
| | C1457 | 1.12 | 54.86 | 23 | 6.84 |
| | C1465 | 13.02 | 66.05 | 15 | 0.85 |
| | C1466 | 1.21 | 72.36 | 23 | 0.00 |
| | C1467 | 2.56 | 130.03 | 22 | 6.00 |
| | C1471 | 12.55 | 78.08 | 17 | 20.96 |
| | C1472 | 24.92 | 57.26 | 13 | 2.66 |
| | C1474 | 20.87 | 61.26 | 14 | 0.00 |

## SM additional References.


1. Ding, C.; Li, T.; Jordan, M.I. Convex and Semi-Nonnegative Matrix Factorizations. *IEEE Trans. Pattern Anal. Mach. Intell.* **2010**, *32*, 45–55.
2. Ortega-Martorell, S.; Lisboa, P.J.G.; Vellido, A.; Julia-Sape, M.; Arus, C. Non-negative Matrix Factorisation methods for the spectral decomposition of MRS data from human brain tumours. *BMC Bioinformatics* **2012**, *13*, 38, doi:10.1186/1471-2105-13-38.
3. Ortega-Martorell, S.; Lisboa, P.J.G.; Vellido, A.; Simões, R. V.; Pumarola, M.; Julià-Sapé, M.; Arús, C. Convex Non-Negative Matrix Factorization for Brain Tumor Delimitation from MRSI Data. *PLoS One* **2012**, *7*, e47824, doi:10.1371/journal.pone.0047824.
4. Delgado-Goñi, T.; Ortega-Martorell, S.; Ciezka, M.; Olier, I.; Candiota, A.; Julià-Sapé, M.; Fernández, F.; Pumarola, M.; Lisboa, P.; Arús, C. MRSI-based molecular imaging of therapy response to temozolomide in preclinical glioblastoma using source analysis. *NMR Biomed.* **2016**, *29*, 732–743, doi:10.1002/nbm.3521.
5. Arias-Ramos, N.; Ferrer-Font, L.; Lope-Piedrafita, S.; Mocioiu, V.; Julià-Sapé, M.; Pumarola, M.; Arús, C.; Candiota, A.P. Metabolomics of therapy response in preclinical glioblastoma: A multi-slice MRSI-based volumetric analysis for noninvasive assessment of temozolomide treatment. *Metabolites* **2017**, *7*, pii: E20, doi:10.3390/metabo7020020.
6. Wu, S.; Calero-Pérez, P.; Villamañan, L.; Arias-Ramos, N.; Pumarola, M.; Ortega-Martorell, S.; Julià-Sapé, M.; Arús, C.; Candiota, A.P. Anti-tumour immune response in GL261 glioblastoma generated by Temozolomide Immune-Enhancing Metronomic Schedule monitored with MRSI-based nosological images. *NMR Biomed.* **2020**, *33*, e4229, doi:10.1002/nbm.4229.
7. Wu, S.; Calero-Pérez, P.; Arús, C.; Candiota, A.P. Anti-PD-1 immunotherapy in preclinical gl261 glioblastoma: Influence of therapeutic parameters and non-invasive response biomarker assessment with mrsi-based approaches. *Int. J. Mol. Sci.* **2020**, *21*, 8775, doi:10.3390/ijms21228775.
8. Ferrer-Font, L.; Villamañan, L.; Arias-Ramos, N.; Vilardell, J.; Plana, M.; Ruzzene, M.; Pinna, L.A.; Itarte, E.; Arús, C.; Candiota, A.P. Targeting protein kinase CK2: Evaluating CX-4945 potential for GL261 glioblastoma therapy in immunocompetent mice. *Pharmaceuticals* **2017**, *10*, 24, doi:10.3390/ph10010024.
9. Ferrer-Font, L.; Arias-Ramos, N.; Lope-Piedrafita, S.; Julià-Sapé, M.; Pumarola, M.; Arús, C.; Candiota, A.P. Metronomic treatment in immunocompetent preclinical GL261 glioblastoma: effects of cyclophosphamide and temozolomide. *NMR Biomed.* **2017**, *30(9)*, doi:10.1002/nbm.3748.
10. Delgado-Goñi, T.; Julià-Sapé, M.; Candiota, A.P.; Pumarola, M.; Arús, C. Molecular imaging coupled to pattern recognition distinguishes response to temozolomide in preclinical glioblastoma. *NMR Biomed.* **2014**, *27*, 1333–1345, doi:10.1002/nbm.3194.
11. Villamañan, L.; Martínez-escardó, L.; Arús, C.; Yuste, V.J.; Candiota, A.P. Successful partnerships: Exploring the potential of immunogenic signals triggered by TMZ, CX-4945, and combined treatment in Gl261 glioblastoma cells. *Int. J. Mol. Sci.* **2021**, *22*, 3453, doi:10.3390/ijms22073453.
12. Chen, D.S.; Mellman, I. Oncology meets immunology: The cancer-immunity cycle. *Immunity* **2013**, *39*, 1–10, doi:10.1016/j.immuni.2013.07.012.
13. von Roemeling, C.A.; Wang, Y.; Qie, Y.; Yuan, H.; Zhao, H.; Liu, X.; Yang, Z.; Yang, M.; Deng, W.; Bruno, K.A.; et al. Therapeutic modulation of phagocytosis in glioblastoma can activate both innate and adaptive antitumour immunity. *Nat. Commun.* **2020**, *11*, 1508, doi:10.1038/s41467-020-15129-8.
14. Karman, J.; Ling, C.; Sandor, M.; Fabry, Z. Initiation of immune responses in brain is promoted by local dendritic cells. *J. Immunol.* **2004**, *173*, 2353–2361, doi:10.4049/jimmunol.173.4.2353.
